# Effectiveness of a large-scale, sustained and comprehensive community health worker program in improving population health: the experience of an urban health district in South Africa

**DOI:** 10.1186/s12960-021-00696-8

**Published:** 2021-12-20

**Authors:** L. S. Thomas, E. Buch, Y. Pillay, J. Jordaan

**Affiliations:** 1 Gauteng Department of Health, School of Public Health, University of Witwatersrand, Bophelo Rd, Prinshof 349-Jr, Pretoria, 0084 Gauteng South Africa; 2 School of Health Systems and Public Health, University of Pretoria and Colleges of Medicine, Pretoria, South Africa; 3 Clinton Health Access Initiative, Pretoria, South Africa; 4 Department of Statistics, University of Pretoria, Pretoria, South Africa

**Keywords:** Community health worker, Population health, Urban, Program effectiveness, Large-scale, Comprehensive, Sustained

## Abstract

**Introduction:**

South Africa is an upper middle-income country with wide wealth inequality. It faces a quadruple burden of disease and poor health outcomes, with access to appropriate and adequate health care a challenge for millions of South Africans. The introduction of large-scale, comprehensive community health worker (CHW) programs in the country, within the context of implementing universal health coverage, was anticipated to improve population health outcomes. However, there is inadequate local (or global) evidence on whether such programs are effective, especially in urban settings.

**Methods:**

This study is part of a multi-method, quasi-experimental intervention study measuring effectiveness of a large-scale CHW program in a health district in an urban province of South Africa, where CHWs now support approximately one million people in 280,000 households. Using interviewer administered questionnaires, a 2019 cross-sectional survey of 417 vulnerable households with long-term CHW support (intervention households) are compared to 417 households with no CHW support (control households). Households were selected from similar vulnerable areas from all sub-levels of the Ekurhuleni health district.

**Results:**

The 417 intervention and control households each had good health knowledge. Compared to controls, intervention households with long-term comprehensive CHW support were more likely to access early care, get diagnosed for a chronic condition, be put on treatment and be well controlled on chronic treatment. They were also more likely to receive a social grant, and have a birth certificate or identity document*.* The differences were statistically significant for social support, health seeking behavior, and health outcomes for maternal, child health and chronic care.

**Conclusion:**

A large-scale and sustained comprehensive CHW program in an urban setting improved access to social support, chronic and minor acute health services at household and population level through better health-seeking behavior and adherence to treatment. Direct evidence from households illustrated that such community health worker programs are therefore effective and should be part of health systems in low- and middle-income countries.

## Introduction

Use of community-based health practitioners such as community health workers (CHW), mid-level workers and similar cadres should be optimized to achieve the agenda of the Sustainable Development Goals [1].

Health services and interventions more frequently reach those with better access to socio-economic resources than those without but who need these services. Ensuring that those who have the greatest need have their health needs met may be difficult to achieve in societies with great wealth inequality such as South Africa. CHW programs improve community equity by being responsive to community needs [2]. They deliver targeted, prioritized services to the most vulnerable of communities. Provision of free health and social services, including home-based care, community mobilization and community participation are important in achieving desired equity goals.

The history and evolution of CHWs over the past decades, in low, middle and high-income countries has demonstrated the impact of selective disease-specific CHW programs in health systems across the world, with only few countries having large-scale comprehensive CHW programs [3, 4].

South Africa introduced a re-engineered large-scale CHW program nationally in 2010–2011, providing comprehensive health and social services to vulnerable communities [5]. CHWs were selected from local unemployed community members and provided with ongoing training. Over 70,000 CHWs, nationally, each supporting approximately 250 households, provided health education, screening for early disease or complications, adherence support and referrals for health and social problems. These CHWs work in teams led by a nurse team leader and are linked to primary health care clinics. They are mainly employed as temporary workers either by the local health district or through a donor agency and paid a small monthly stipend [6].

Where large-scale CHW programs provide a sustained and comprehensive range of services [7], it is claimed they can strengthen primary health care within health systems and play an important role in achieving desired health outcomes. However, there is little good-quality evidence in this regard, especially in real-world settings. One of the few countries that has conducted evaluations of their large- scale CHW programs is Brazil, where Family Health Teams support designated households. Household surveys demonstrated improvements in health status of the population [8].

Our study is conducted in the Ekurhuleni Health District, a densely populated urban district in South Africa (Appendix 1). Its approximately four million people, live in one million households [9], mostly in poor areas that rely on the public health system for care. The population includes undocumented migrants.

The CHW program in Ekurhuleni started in 2010 and has since grown to over 1100 permanently employed CHWs providing comprehensive care and support to approximately one million people in 280 000 vulnerable households. This is a large-scale program. The CHWs earned a monthly stipend of USD 232 at the time of study. Six to ten CHWs form a team led by a nurse. The role of the nurse is to plan, manage, support and supervise the work of the CHWs in the team. The CHWs routinely visit the same allocated households, building relationships over long periods of time. They conduct early screening and referrals for pregnant women through urine tests and assess children for immunization status, pneumonia, diarrhoea and malnutrition. They screen for HIV, TB, hypertension and diabetes by asking about signs and symptoms and using digital blood pressure and glucose machines. They ask women over the age of 35 years about pap smears with a view to referral to screen for cervical cancer. Health education for all, adherence support for chronic care, defaulter tracing for all the above conditions and psycho-social support are also part of the Ekurhuleni CHW program. Tracing of defaulters takes place in households allocated to CHWs as well as households outside of the allocated areas. Defaulters are those who fail to turn up for test results, diagnosis or follow-up treatment. According to the data collected by the Ekurhuleni district CHWs, these comprehensive, sustained activities have resulted in improved household referrals, reporting to clinics and access to health care [7].

However, it is also important to determine if such large-scale, sustained and comprehensive activities make a difference in household health outputs and outcomes. There is inadequate evidence on the effectiveness of large-scale comprehensive CHW programs in improving population health, especially in Africa [10, 11], defined by its ability to have a meaningful effect under normal conditions [12]. Our study aims to address this gap by answering whether the CHW program in Ekurhuleni did what it intended to do in contributing towards improvements in health and social outputs and outcomes in CHW supported households.

## Methods

This study determined if CHWs were effective in improving household (HH) health. We explored measures of CHW effectiveness by looking at social and health outputs and outcomes.

### Study design

The study is based on an analysis comparing 417 intervention households exposed to CHWs with a control group of 417 households not exposed to CHWs, in Ekurhuleni, South Africa. Household interviews were conducted between April and June 2019.

Health and social support output measures were explored through reported perceptions of access to social services, early antenatal and child health services and chronic disease care (HIV, TB, hypertension and diabetes), improvements in care and knowledge of common health conditions and on healthy behavior (diet, exercise, hand washing). Self-reported health outcome measures for maternal, child health issues and chronic disease were also explored. For the purposes of this study, the social support activities are defined as support in getting a birth certificate, an identity document for adults, social grants and food parcels.

### Sampling of households

The district is divided into East, North and South sub-districts and vulnerable households were selected from all three. In Ekurhuleni, properties valued at less than USD 9415 were considered indigent/vulnerable. The six district intervention sites in Ekurhuleni that had more than 60% CHW team coverage, two from each sub-district, with approximately 56 000 households in total were identified. Intervention households were proportionately selected from these areas. Assuming a 95% confidence level, 5% margin of error and 50% response distribution, using Raosoft sample size calculator,^1^ a sample size of 381 households was representative of this population. A 5% buffer was added to cover for replacement households or incomplete surveys, so the sample size was approximated to 400 intervention households/HH. A map divided into small areas was used and each day, a random spot on the map was selected as a starting point. The field workers went to every fifth household supported by CHWs until the required number of households were reached.

400 control households in Ekurhuleni were chosen randomly in the same way; situated in similar areas, but where CHW teams were not working.

Eleven retired nurses proficient in the locally used language were trained as fieldworkers to undertake the household interviews.

The questionnaires were piloted in an area in the same district in a site not included in the study and fieldworkers were asked to provide feedback on their experiences in the field. The questions in the tools were easily translated and understood, so no major changes had to be made.

Inclusion criteria included that the household member interviewed had resided in the area for 18 months or longer and that each household had at least one vulnerable member (a pregnant woman, child under five, an elderly person, or a household member with a chronic disease). Intervention households had to have an allocated CHW, and controls not. The household head or the person who knew about other members was interviewed. 417 intervention and control households each were interviewed, but due to some missing data, approximately 400 households in each group were available for analysis.

### Analysis

Frequencies of the socio-economic and demographic variables were conducted. For the categorical data, we mitigated for possible confounders by correcting the data for socio-economic and demographic differences between the two groups (Tables 1 and 2). In order to compare equitable access to social services, we excluded non-South African and non-indigent households from the analyses; we also corrected where relevant for gender and age in HIV status, family planning, immunization and chronic diseases (Table 3). We conducted bivariate analysis using Pearson Chi-squared tests. 2 × 2 tables were also used to compare the effect of the exposure to CHW teams in intervention households (Table 4). In cases where the Pearson’s Chi-square test was significant, the cell standardized residuals, expected and observed values were investigated to establish which cells in the cross-tables contributed most to the significant associations between two variables.

**Table 1:** Socio-economic and demographic measures of ALL study households

Socio-economic and demographic measures	Responses	*n* = number (%)		Pearson's Chi-square test (*p* value)
		Intervention households *n* = 417	Control households *n* = 417	
Gender of client interviewed	Male	69 (17%)	73 (18%)	*p* = 0.677
	Female	348 (83%)	341 (82%)	
	Total	417	414	
Age of respondents	< 18 years	1 (0%)	2 (0%)	***p* < 0.001.** Intervention households had more Interviewed clients over 50 years of age
	18–25 years	24 (6%)	23 (6%)	
	26–50 years	183 (44%)	263 (63%)	
	> 50 years	204 (50%)	127 (31%)	
	Total	412	415	
Head of household interviewed?	Yes	340 (82%)	328 (80%)	*p* = 0.529
	No	77 (18%)	83 (20%)	
	Total	417	411	
SA citizen	Yes	393 (95%)	352 (85%)	***p* < 0.001.** More households with SA citizens in Intervention group
	No	19 (5%)	63 (15%)	
	Total	412	415	
Estimated value of property (indigent households = < USD 9715) USD: United States dollar	< USD 9715	312 (76%)	381 (92%)	***p* < 0.001**. Intervention group had fewer indigent households
	> USD 9715	101 (24%)	34 (8%)	
	Total	413	415	
How long had they resided in the area?	1–3 years	41 (10%)	49 (12%)	*p* = 0.281
	4–5 years	39 (9%)	28 (7%)	
	> 5 years	335 (81%)	339 (81%)	
	Total	415	416	
Own the home they live in?	Yes	330 (79%)	330 (79%)	*p* = 0.997
	Not sure	9 (2%)	9 (2%)	
	No	77 (19%)	78 (19%)	
	Total	416	417	
Total household income per month	< USD 130	286 (70%)	339 (80%)	***p* < 0.001.** Household monthly income was higher in the intervention group
	USD 130–324	99 (24%)	71 (17%)	
	> USD 324	26 (6%)	5 (1%)	
	Total	411	415	
Age distribution of household members	Child under 5 years	199 (13%)	316 (19%)	Proportionately more younger members in control households and > 60 years in intervention households
	5–15 years	343 (23%)	391 (23%)	
	15–60 years	735 (48%)	866 (52%)	
	> 60 years	243 (16%)	102 (6%)	
	Total	1520	1675	

Statistically significant *p* values (< 0.05) were also in bold

**Table 2:** Corrected socio-economic and age variables in South African indigent households

General socio-economic and demographic measures	Variables	*n* = number (%)		Pearson's Chi-square test (*p* value)
		Intervention households	Control households	
Adjusting for socio-economic differences	Total no. of households studied	417	417	
	No. of SA citizen households	393/412 (95%)	352/415 (85%)	***p* < 0.001** Significantly more SA citizen households in intervention group
	No. of indigent South African households	292/393 (74%)	318/352 (90%)	***p* < 0.0001** Significantly fewer indigent households in intervention group
Age of respondents in indigent South African households	18 to 50 years	155/292 (53%)	219/318 (69%)	***p* = 0.02**. More respondents > 50 years in intervention group
	> 50 years	133/292 (46%)	96/318 (30%)	
Age of members in indigent South African households	Children < 5 years	150 (14%)	236 (18%)	*p* = 0.122
	Children 5–15 years	242 (23%)	299 (23%)	
	15 to 60 years	498 (48%)	681 (53%)	
	> 60 years	152 (15%)	69 (5%)	
	Total	1042	1285	

Statistically significant *p* values (< 0.05) were also in bold

**Table 3:** Health and social outputs and outcomes in South African indigent households*

Health and social measures of change	Intervention households *n* (%)	Control households* n* (%)
Health and social outputs		
Access to social support services		
Households that received food parcels	24/292 (8%)	23/318 (7%)
Members > 60 years that receive an old age grant	81/152 (53%)	38/69 (55%)
Members < 15 years that receive a child care grant	311/392 (79%)	389/535 (73%)
Members of all ages that receive a disability grant	21/1042 (2%)	28/1285 (2%)
Members < 15 years that have a birth certificate	387/392 (95%)	461/535 (79%)
Members > 15 years that have a national identity card	635/650 (95%)	659/750 (71%)
Self-reported health seeking behavior		
Household accessing clinic care in past 12 months	127/292 (43%)	226/318 (71%)
Family planning in 18- to 50-year-old female household respondents	92/135 (68%)	143/186 (77%)
Respondents health awareness and knowledge (based on knowledge score)**		
Awareness of HIV status in 18- to 50-year-old household respondents	153/155 (99%)	213/219 (97%)
Knew signs of TB	287/292 (98%)	309/318 (97%)
Knowledge on hypertension in household respondents	272/288 (94%)	293/315(93%)
Knowledge on diabetes in household respondents	246/288 (85%)	273/315 (87%)
Knew importance of child immunization in household respondents	270/288 (94%)	304/315 (97%)
Health outcomes		
Self-reported morbidity (from household interviews)		
Number of members 15–60 years with chronic condition diagnosed and on treatment	123/498 (25%)	101/698 (14%)
Number of members > 60 years with chronic condition diagnosed and on treatment	81/152 (53%)	28/69 (41%)
Households with (any) chronic condition well controlled over last 12 months	220/292 (75%)	210/318 (66%)
Number of members < 5 years immunized	110/150 (73%)	160/236 (72%)
Number of pregnant women who accessed early antenatal care (< 20 weeks)	8/11 (73%)	19/31 (61%)

*Shaded areas represent more than 5% differences between the two groups of households

** Respondents were asked a specific question

**Table 4:** Comparison of health and social outputs and outcomes in South African indigent households

Intervention vs control households		Odds ratio (2 × 2 table)*	2-tailed *p* value
Health and social outputs			
Access to social support services	Received any social grant (old age, child care and disability grant)	**1.3**	**0.004**
	Had SA birth certificate or ID	**4.9**	**0.001**
Self-reported health seeking behavior	Use of family planning	0.5	**0.01**
	Improvements in early access to health care in last 12 months	**3.93**	0.169
Health awareness and knowledge (based on knowledge score)	HIV status	**1.79**	0.481
	Tuberculosis	**1.62**	0.43
	Hypertension	**1.28**	0.47
	Diabetes	0.92	0.73
Health outcomes			
Self-reported morbidity (from household interviews)	Chronic condition diagnosed and on treatment in members	**2.4**	**0.002**
	Chronic condition well controlled in last 12 months in households	**1.6**	**0.0001**
	Immunized child	**4.3**	**0.039**
	Early antenatal care (< 20 weeks)	**2.3**	0.33
	Healthy children	0.3	**0.0001**
	Safe pregnancy	0.07	**0.0001**

Statistically significant *p* values (< 0.05) were also in bold

*Where odds ratios > 1 (in bold), it is in favour of intervention households; where odds ratios < 1, control households did better

## Results

This section details the health and social outputs and health outcomes in 800 households, comparing the findings between intervention and control households.

Table 1 shows that female adults, mostly South African, headed 80% of the households in both groups. 80% of those interviewed had resided in the area for longer than 5 years.

The intervention and control households had a few differences. Control households had more indigent and fewer South African households and proportionately more children under 5 years of age, while intervention households had proportionately more members over 60 years.

Even though the majority of all households were poor, the results have been corrected for all the differences mentioned. We focused on South African indigent households and used the age of respondents and household members to compare output and outcome variables (Table 2). Age, gender, income and being a foreigner were therefore no longer confounders.

Table 3 shows the proportionate similarities and differences between intervention and control households in the various age groups. Fewer control household members had a birth or identity document. 71% of controls appeared to access clinic care compared to 43% of intervention households. Control households also used family planning more. Households in both groups had similar and high levels of health knowledge and awareness. Both groups were aware of their HIV status and knew the signs of tuberculosis. Most intervention and control households were aware of risk factors for common chronic conditions such as diabetes and hypertension. The majority of households were aware of the importance of child immunization. Intervention households reported improved early antenatal care access and chronic disease diagnosis and control; both in 15–60 (25%) and > 60-year olds (53%). Intervention households had more children on child care grants. Both groups had similar proportions of members on old age grants and children reported immunized.

In comparing intervention households to control households (Table 4), odds ratios > 1 were in favour of intervention households for 10 of the 14 variables studied, with five statistically significantly. In addition, odds ratios were in favour of controls for four variables, three being significant. Intervention households were more likely to have received better overall social support. They were 1.3 times as likely to have received any social grant and five times more likely to have a birth certificate and/or identity document for relevant members than control households.

Health seeking behavior of those in intervention households, as stated, was better, intervention household members were four times more likely to demonstrate improved access to health care over the past year.

Health outcomes with respect to morbidity was compared between the intervention and control households. Morbidity was reported as better managed in intervention households. Where an intervention household had a member with a chronic condition, they were 2.4 times more likely to have been diagnosed and put on treatment and twice as likely to be well controlled on that treatment. Control households were more likely to report healthier children and safer pregnancy. However, a child from an intervention household was four times as likely to be immunized. A pregnant woman living in an intervention household was 2.3 times as likely to seek antenatal care early.

## Discussion

This study has shown that a *large-scale*, *sustained* and *comprehensive* CHW program improved health and social outputs and health outcomes in a vulnerable *urban* population. This is important for South African policy-makers as they transform the health system to improve population health outcomes for all and this study adds new knowledge supporting the use of CHWs in vulnerable urban areas.

**Fig. 1:**
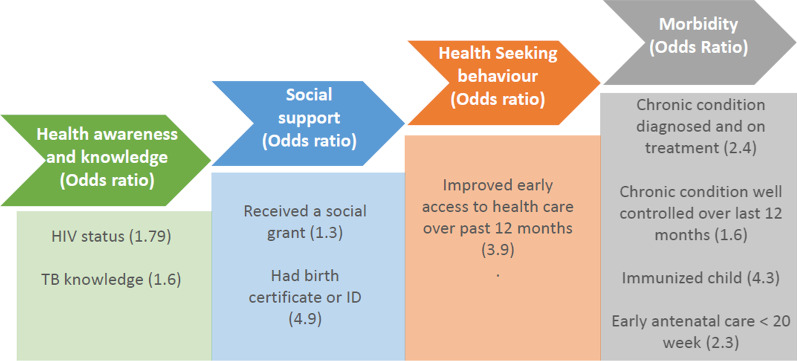
Summary of changes in intervention households

We compared households supported by CHWs to those not supported by CHWs and corrected for socio-economic and demographic differences. Despite similar, high health education knowledge in both groups of households, possibly because of the choice of too simple knowledge measures in our tool, households supported by CHWs had better improvements in health and social outputs and outcomes (Fig. 1).

Findings from the household interviews in our study showed that female-headed households were the majority in both intervention and control households; and nearly all were from a low socio-economic stratum. Female headed households [13] while commonly associated with poverty in poorer, rural areas of South Africa; are also in poor urban settings as seen in our study sample. The property value and monthly income was reported as low in both intervention and control households. This was not surprising since vulnerable areas in the district were selected for the study.

When comparing social outputs, access to social services such as birth certificates and identity documents was significantly better in intervention households. Identification is critical in giving people access to a broader range of services such as welfare grants and food parcels. When looking at access to any welfare grant; intervention households again fared significantly better. We infer that this was due to the efforts of the CHWs in checking children and adults without these documents and referring them to social services. Enabling social support services in vulnerable households is an important component of the CHW program in the Ekurhuleni health district [7] and an opportunity to build trust and credibility between the CHW and households, which over time, can influence other changes.

Comparing health outputs, many more in control households were younger, compared to an older demographic in intervention households, so they were more likely to use family planning, have young children and be aware of their HIV status. 71% of controls indicated they accessed early care in local clinics compared to 43% in intervention households, possibly indicating that control households went to clinics for even simple and minor issues that CHWs were able to resolve in the households they visited, resulting in members of intervention households being less likely to need to go to the clinic. In intervention households we found that they too accessed clinic care, but more so when actually sick and/or when referred by their CHW.^[7]^All households analyzed were South African and indigent and there were no barriers to access to clinic care in both groups. We postulate that by managing issues better in the households they cover, the CHW program in Ekurhuleni contributes to improved appropriate health seeking behavior by intervention household members and less crowding in local clinics [14].

Health awareness was generally good in both household groups in this poor urban community. Health information from sources such as newspapers, radio, television and information from local health facilities appear to have reached both intervention and control households [15, 16]. We asked simple easily understood multiple choice and true/false questions to assess knowledge of tuberculosis, hypertension, diabetes, family planning and hygiene. We asked about the signs and symptoms of tuberculosis, whether salty food contributed to hypertension, link of obesity to diabetes, use of family planning and when to wash hands. These questions, while seemingly suitable, may not have been discerning enough to identify a difference in knowledge between intervention and control households.

When comparing health and disease outcomes, controls may have subjectively reported healthier children and safer pregnancy; there may have been missed opportunities in early identification of problems which were identified by CHWs in the intervention households, i.e., the finding that more control children and pregnant women were viewed by household heads to be healthy may mean a higher threshold of acceptance of symptoms and reluctance to seek care in households who do not benefit from visits of CHWs. Intervention households fared better in access to early antenatal care and significantly better in child immunization, as well as early diagnosis, treatment and control of chronic diseases. We found that even with reasonable health education levels and use of available services, this alone was not sufficient to improve health and social outputs in control households, leading us to infer that the CHWs played an important role in changing reported health outcomes in intervention households.

The South African immunization schedule^2^ includes pneumococcal, rotavirus and haemophilus influenza. Improved access to immunizations is likely to have been a contributor to reduced child morbidity in intervention households. Indeed in one of our previous studies [7] we showed that CHW efforts reduced un-immunized children and those with diarrhoea, pneumonia and malnourishment, as reiterated elsewhere [17]. Mothers of sick babies or unimmunized children were more likely to respond positively to their CHW and be referred to care; with uptake of such referrals generally high as evidenced in our own [7] and other studies [18]. Access to early antenatal care was considered a health outcome indicator because it provided opportunities to identity challenges early in pregnancy such as nutritional deficiencies, HIV, hypertension and/or other risks to the pregnancy and provide the care needed to ensure a good pregnancy outcome for mother and baby. Early antenatal screening and care was a critical part of the activities of the district CHW program [7] and results show improved early antenatal screening in intervention households compared to controls. The CHW program therefore improved child and maternal health outputs and outcomes in the intervention households. These are important outcome measures of population health.

Intervention households did better with regard to chronic disease control, including HIV, TB and non-communicable diseases. This shows that CHWs in Ekurhuleni play a valuable role in promotion of health and prevention of disease. This is in line with the literature supporting the role of CHW as a health educator and community advocate [19]. While there are a limited number of studies exploring the effectiveness of CHW programs on preventative programs specifically, there is moderate evidence of benefit to maternal and child health programs [20]. Our study showed that secondary prevention was also possible; morbidity (or illness) in intervention households was generally identified earlier, with better access to care and importantly better retention in care for chronic disease, compared to control households. This again can be related to the support in the households over the years by CHWs in concordance with studies showing that relationships built over time [21] influence behavior change and health outcomes [22, 23]. Households reported to clinics as recommended by their CHWs and accessed care earlier for all conditions. This value is important when one considers the increasing burden from non-communicable diseases [24]; CHW programs have the potential to improve early detection and adherence to care for all chronic diseases [25]. The World Health Organization calls for more global evidence on the role of CHWs in non-communicable diseases [10], and our study adds to new knowledge that demonstrates the contribution of CHWs in supporting the control of non-communicable and other chronic diseases through improved retention in care.

A challenge in chronic disease control faced by CHW teams is dealing with large numbers of defaulters in the health system. In South Africa, chronic diseases include HIV and TB; externally funded donors play a role in helping the state to address this [26]. Improving adherence support in households, with support from CHW programs, could reduce the number of defaulters from these conditions in the long term [27]. Increased health awareness, active case finding, healthy behavior change and good adherence in CHW supported households, as shown in our study, changes this and reduces the number of defaulters. This requires frequent CHW interactions over long periods of time. Improvements in population health require long-term strategic public health sector and donor investments. Long-term donor commitments [28] should thus be considered in engagements between the state and donors [29, 30]. Through large-scale, long-term, comprehensive CHW programs such as the one in Ekurhuleni, South Africa should mobilize more community-based resources to reinforce healthy behavior change and better adherence support in communities.

The CHW program in Ekurhuleni commenced in 2010–2011; with new teams added each year. The program was sustained in the district for almost a decade; resulting in adequate time for CHWs in the program to build relationships and influence health outputs and outcomes in these households. Most of those interviewed in the households had resided in the study areas for 5 years or longer [31]. This meant that the intervention household members had been in the area long enough to judge if the CHWs supporting them had made an impact.

Ekurhuleni CHW teams influenced health outputs and outcomes in vulnerable households thus improving population health. State funding challenges over the years has constrained expansion of the number of teams to increase district CHW household coverage. Furthermore, the district population has increased year on year, reducing proportionate CHW team coverage. Our study demonstrated the effectiveness of CHW household coverage on improving population health over time in vulnerable communities. This is a relatively low-cost intervention ensuring promotion of health and prevention and control of disease; the fundamentals of primary health care. Investing more in such comprehensive CHW programs [32] in South Africa could lead to scale-up services in more vulnerable households, reducing state costs associated with disease morbidity and mortality over the long-term.

## Study limitations

CHW team coverage of communities determined selection of intervention households. Since this was a real-world study setting, households could not be randomly assigned. Possible confounders were mitigated by selecting vulnerable households in all three Ekurhuleni sub-districts which were similar socio-economically and correcting for the differences, thereby matching intervention and control households.

Our study findings are based on the information from one household member; there could have been recall bias in what was remembered and known about other household members as well as socially desirable responses. However, trained fieldworkers ensured that the interviewed member was one who knew the most about rest of the household and recall bias would affect both intervention and control households similarly.

The fieldworkers identified a number of households that could have been migrants, possibly undocumented. We considered if this could be a confounder, but felt this was not the case as both control and intervention households had resided in the area for several years, spoke the local language, had similar high health knowledge and had equal access to public health services.

## Conclusion

Intervention households supported by CHWs were better at accessing care, being diagnosed early, staying on treatment and accessing social support compared to control households for chronic diseases and mother and child health needs. The CHWs played an important role in providing social support services; which would help to partly address some of the social determinants of health in households and foster trust and credibility in the relationship. The reinforcement of health messages, reminders and prompts by CHWs is likely to have influenced health seeking and lifestyle behavior change in households over time. The sustained relationships built between the CHW and the intervention households likely contributed to the differences in health outcomes found.

Our study adds valuable new knowledge demonstrating that *large-scale*, *sustained* and *comprehensive* CHW programs in *urban* settings are important in achieving universal health coverage and the sustainable development goals of lower- and middle-income countries, especially in Africa.

## Recommendations

Our study has shown that CHW programs supporting households well yield improvements in population health. South Africa with its rising burden of chronic diseases and unmet need should increase its investment in such programs; this is an important consideration for district resource planning.

Public health interventions utilize public funds and must yield results; these can be shown through effectiveness studies, such as our study, demonstrating the potential in a real-world setting. Additional studies on the impact of CHW programs are required. More of such studies should be encouraged.

## Figures and Tables

**Fig. 2:**
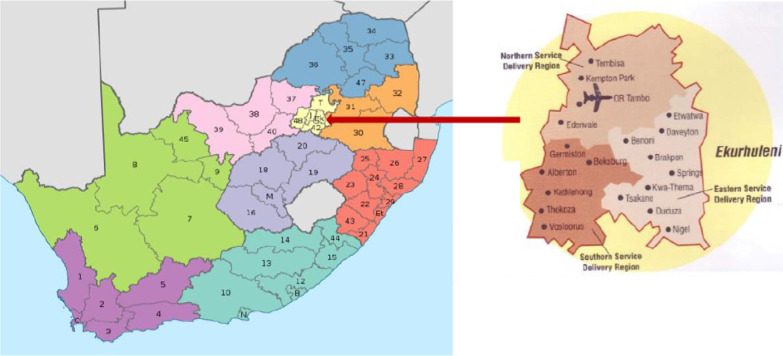
Map of South African health districts and Ekurhuleni specifically. Source: https://en.wikipedia.org/wiki/Districts_of_South_Africa & Ekurhuleni Integrated Development Plan Booklet 2004

## Data Availability

On completion of related studies.

## Appendix 1

See Fig. 2.

## Footnotes

1 http://www.raosoft.com/samplesize.html

2 http://www.health.gov.za/wp-content/uploads/2020/11/epi-schedule.pdf
